# Favism‐induced methemoglobinemia in a G6PD deficient male with a subsequent hemolytic cascade, a therapeutic challenge: Case report and review of literature

**DOI:** 10.1002/ccr3.3941

**Published:** 2021-02-20

**Authors:** Fateen Ata, Saad Javed, Bassam Muthanna, Ines dakhlia, Ammara Bint I Bilal, Motwakil Musa, Mashuk Uddin, Mohamed A. Yassin

**Affiliations:** ^1^ Department of Internal Medicine Hamad Medical Corporation Doha Qatar; ^2^ Department of Internal Medicine Jinnah Hospital Allama Iqbal Medical College Lahore Pakistan; ^3^ Department of Radiology Hamad Medical Corporation Doha Qatar; ^4^ Department of Hematology National Center for Cancer Care & Research Hamad Medical Corporation Doha Qatar

**Keywords:** fava beans, Favism, G6PD, glucose‐6‐phosphate dehydrogenase, Hemolysis, methemoglobinemia

## Abstract

The co‐occurrence of acute hemolysis and methemoglobinemia secondary to favism in G6PD deficient individuals is rare. Identifying it promptly is of high clinical significance as treating methemoglobinemia (with methylene blue) can worsen hemolysis.

## Graphical abstract

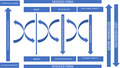

## 1 INTRODUCTION

We report a 56‐year‐old man with methemoglobinemia and hemolytic anemia, secondary to fava bean ingestion. Methylene blue administration worsened the hemolysis as he was G6PD deficient but not diagnosed before. We have discussed the mechanism of hemolysis in such patients and the management of such cases.

Hemolytic anemia, a form of anemia that causes premature rupture of erythrocytes, accounts for five percent of anemias.^1^ Glucose‐6‐phosphate dehydrogenase (G6PD) deficiency is a well‐known cause of hemolysis and currently affects around 400 000 000 individuals globally. It has a notable prevalence in African, Asian, and Mediterranean countries.^2^ Favism is a common trigger of oxidative stress in G6PD deficient people, which can lead to hemolysis. Additionally, fava bean ingestion can cause methemoglobinemia.^3^ Methemoglobin is an abnormal variation in the hemoglobin in which the ferrous (Fe2^+^) iron in heme is oxidized to the ferric (Fe3^+^) state. The condition is usually acquired, secondary to oxidative stress in the body such as favism or infections, but can rarely be congenital.^4^ The first‐line treatment for methemoglobinemia is methylene blue. However, in G6PD deficient patients, methylene blue can potentiate hemolysis because of its oxidative effects.^3^ It is vital to take a detailed history of patients presenting with hemolysis to identify the potential causes and avoid any additional oxidative stress.

## 2 CASE PRESENTATION

A 56‐years‐old Qatari man, known to have type‐II diabetes mellitus and hypertension, presented with a 5‐day history of progressive dyspnea and dizziness. He also had a 3‐day history of mild hematuria and one episode of minimal nonbloody vomiting. The patient had no recent infection and no exposure to new medications. He is married (nonconsanguineous), a smoker (5 cigarettes per day) but nonalcoholic with no history of illicit drug use.

**FIGURE 1:**
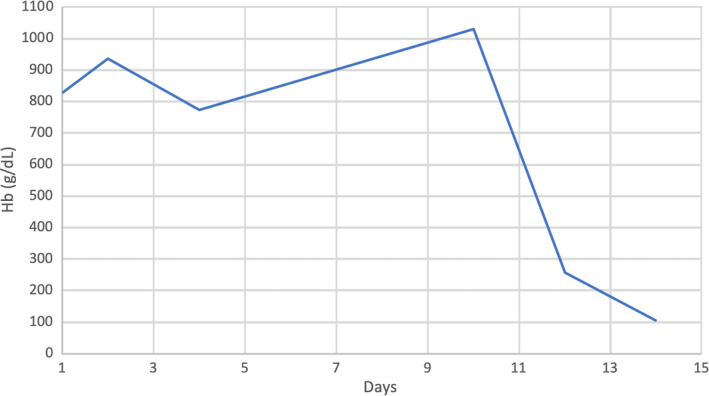
Trend of hemoglobin of the patient throughout the hospital stay

Upon examination, he was vitally stable (afebrile, Blood pressure 136/76 mm Hg, heart rate 93 beats per minute) other than an oxygen saturation (SPO_2_) of 70% on room air. On examination, he had pallor and jaundice. The rest of the physical examination was unremarkable. Arterial blood gas (ABG) analysis revealed SPO^2^ of 101% and a methemoglobin (MetHB) level of 5.6% (Table 1). The patient was initiated on supplemental oxygen, but his SPO_2_ remained low. Because of a high MetHgb level, a provisional diagnosis of methemoglobinemia was made, and he received methylene blue intravenously (IV) 80 mg while in the emergency department. A complete blood analysis revealed low hemoglobin (Hgb) of 9.9 gm/dL, secondary to hemolysis (Table 1, Figure 1). Chest x‐ray and electrocardiogram were unremarkable. A urine dipstick analysis did not reveal significant blood or protein.

**TABLE 1:** Clinical features of the patient

Hematological parameters	Patient code	Normal range
WBC count	31000	4.5‐11 *10^3^/μL
Platelets	515	150‐400 ×10^3^/μL
MCV	98.7	83‐101 fL
MCHC	32.9	31.5‐34.5 gm/dL
RI	2.47	>2
PO_2_ on ABG	524	83‐108 mm Hg
MetHgb	5.6%	<1.6%
Indirect bilirubin	60	<13 μmol/L
LDH	Hemolyzed	135‐225 U/L
Haptoglobin	15	30‐200 mg/dL
G6P6D quantity	23	224‐517 mU/10^9^RBC
PS	Bite cells, blister cells, left shift	–
Carboxy Hgb	3.3%	Nonsmoker: <1.5% of Hgb Smokers: <1.5%‐5.0% of Hgb
HgbE	HBA:96, HBA2:2.6, HBS:0, HBF:1, HBH: absent	HBA:95.8‐98, HBA2:2‐3.3, HBS:0, HBF:0‐0.9, HBH: absent
CRP	67	0‐5 mg/L

Abbreviations: CRP, c‐reactive protein; Hgb, hemoglobin; HgbE, hemoglobin electrophoresis; LDH, lactate dehydrogenase; MCHC, mean corpuscular hemoglobin concentration; MCV, Mean corpuscular volume; MetHgb, methemoglobin; PS, peripheral smear; RI, reticulocyte index; WBC, white blood cell.

A repeated Hgb level after 24 hours showed a further drop to 7 gm/dL. As he was symptomatic, two units of packed red blood cells (RBC) were transfused. Unexpectedly, his Hgb continued to drop further (Figure 1). Ongoing hemolysis was evident, and a detailed history was retaken to identify the cause of hemolysis. The patient revealed an intake of large amounts of fava beans, which preceded his symptoms. He had a history of eating fava beans in small amounts before without experiencing any symptoms. However, this time, the intake was considerably larger (six fava beans containing sandwiches). Because of suspicion of favism‐induced hemolysis, a G6PD level was sent, which came low (Table 1). At this point, he was diagnosed with G6PD deficiency, aggravated by the ingestion of a large number of fava beans. His hemolysis was worsened by methylene blue, which was evident by a progressive drop in Hgb.

The patient was kept in the medical ward under close observation. He received a total of 3000 mg of IV Vitamin C in two divided doses. After 2 days, his SPO2 improved to 100% on room air, and Hgb improved gradually to 11 gm/dL on the fifth day (Figure 1). He was discharged as he became asymptomatic on day five with a follow‐up in the acute medical assessment clinic.

## 3 FOLLOW‐UP

Follow‐up lab work showed near‐normal Hgb (12 gm/dL), normal bilirubin, and liver enzymes. The patient did not have any residual dyspnea. There was no jaundice, and his hematuria had resolved. He was discharged from the clinic with clear instructions about his diagnosis. He was also counseled to avoid fava beans in the future. He was using aspirin as primary prevention of cardiovascular disease and was advised to discontinue it to avoid oxidative stress. The patient was offered genetic testing of G6PD at the follow‐up visit in the genetics clinic, which he refused.

## 4 DISCUSSION

Glucose‐6‐phosphate dehydrogenase (G6PD) deficiency is the most prevalent blood cell disorder in humans.^5^ It is an X‐linked genetic disorder caused by a chromosome X defect (band X q28).^6^ It is usually diagnosed when patients present with signs and symptoms of hemolytic anemia, secondary to oxidative stress. It is usually triggered by infections, fava beans, and certain medications.^7^ Various screening tests are available to detect G6PD deficiency, and the diagnosis is usually confirmed by quantitative measurement of nicotinamide adenine dinucleotide phosphate (NADPH).^8^ The primary differential diagnoses of G6PD deficiency are other causes of hemolysis. In malaria‐endemic areas, malaria is one of the differentials of G6PD hemolysis. Other causes of inherited and acquired hemolytic anemias need to be considered while diagnosing G6PD deficiency‐induced hemolytic anemia.^9^ Genetic testing can be carried out via polymerase chain reaction; however, it is usually not routinely performed and limited to challenging situations and atypical presentations.^9^ Treatment of acute episodes of hemolysis is by transfusion and, more importantly, eliminating the cause of oxidative stress.^10^

On the other hand, methemoglobinemia is a hemoglobin disorder where ferrous (Fe2^+^) iron in heme is oxidized to the ferric (Fe3^+^) state. It is usually acquired, secondary to oxidative stress in the body, but can rarely be congenital.^4^ Physiologically, various enzyme systems such as NADH methemoglobin reductase, NADPH methemoglobin reductase, ascorbic acid, and glutathione reductase systems keep a check on the accumulation of methemoglobin in the blood.^11^ However, there are instances where these mechanisms are insufficient to counter the conversion of hemoglobin to methemoglobin, consequently promoting an oxidative state in the body. This can be either due to the overproduction of methemoglobin or under conversion to hemoglobin due to unavailable enzyme mechanisms. The former can be secondary to exposure to certain drugs, chemicals, or food items but can sometimes be hereditary.^12^ The inability of enzyme systems to counteract methemoglobin can be secondary to enzyme deficiencies, such as G6PD deficiency.

**FIGURE 2:**
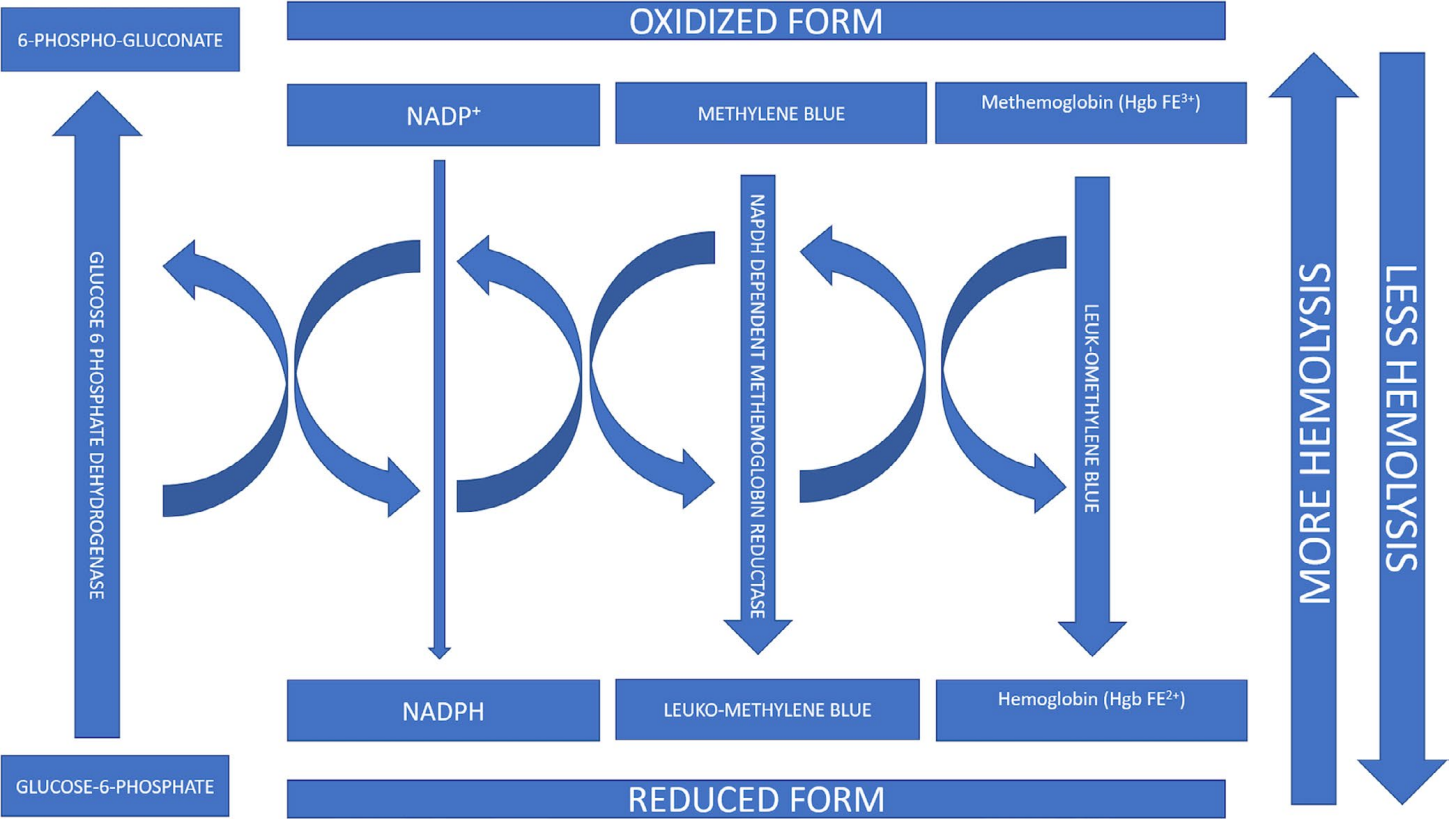
Mechanism of reduction of methemoglobin to hemoglobin by methylene blue; concept taken from Percy MJ et al^15^

Usually, the patients typically have a low SPO^2^ on pulse oximeters but a falsely high SPO^2^ on arterial blood gasses (ABG).^13^ The treatment depends on the level of methemoglobin in the body and symptoms. The first step is to remove any possible precipitator if present immediately. The treatment of choice for symptomatic or asymptomatic patients with a level of methemoglobin >30 percent is methylene blue (1‐2 mg/kg).^14^ Methylene blue is reduced to leuko‐methylene blue via NADPH‐dependent methemoglobin reductase. This, in turn, reduces methemoglobin back to hemoglobin, hence correcting the abnormality ^15^ (Figure 2).

Rarely, patients can present with co‐occurrence of methemoglobinemia and G6PD deficiency.^3^, ^16^, ^17^, ^18^, ^19^ In such cases, extreme caution is required while administering methylene blue as they do not have sufficient NADPH levels to reduce it. Otherwise, a cascade of oxidative hemolysis ensues secondary to underlying G6PD deficiency, resulting in a vicious cycle of further methemoglobinemia.^11^

The most frequent cause of this co‐occurrence reported in the literature is the ingestion of fava beans, which can induce methemoglobinemia and potentiate G6PD deficiency simultaneously.^3^, ^16^, ^17^, ^18^, ^19^ All the reported cases in the literature are male, with a median age of 6 years (range 1‐56). All of them were newly diagnosed with G6PD deficiency upon presentation with MethHgb. Median Hgb was 8 gm/dL (4.6‐9.9) and median MetHgb was 8% (5.6‐35). One patient (our patient) received methylene blue, and 3 received Vitamin C. All of them recovered and were discharged. Our patient was also male and had taken a full meal consisting of fava beans before presenting. Although his methemoglobin level was 5.6 percent, he was given methylene blue due to his symptoms, which worsened his hemolytic anemia (Table 2).

**TABLE 2:** Reported cases of methemoglobinemia and G6PD deficiency secondary to favism

Case number	Patients' age, sex Nationality	G6PD deficiency	Type	Hgb (gm/dL) presentation/lowest	MetHgb	Methylene blue	Vit C	Outcome
Case 1 ^16^	43 y, male Albanian	New diagnosis	NA	8/NA	8%	No	Yes	Discharged
Case 2 ^18^	6 y, male Algerian	New diagnosis	G6PD^376G/202A^	9.2/6	7.6%	No	No	Discharged
Case 3 ^19^	30 y, male Nepalese	New diagnosis	NA	8.4/5.9	35%	No	Yes	Discharged
Case 4 ^3^	1 y, male Afghan	New diagnosis	NA	6.2/6.2	6.2%	No	No	Discharged
Case 5 ^17^	1 y, male Iraqi	New diagnosis	NA	6.9/NA	11.4%	No	No	Discharged
Case 6 ^17^	6 y, male Iraqi	New diagnosis	NA	4.6/NA	14.9%	No	No	Discharged
Our case	56 y, male Qatari	New diagnosis	NA	9.9/6.5	5.6%	Yes	Yes	Discharged

Abbreviations: G6PD, Glucose‐6‐phosphate dehydrogenase; Hgb, Hemoglobin; MetHgb (normal 0%‐1.5%), Methemoglobin.

Interestingly, our patient had a history of favism in the past without developing any symptoms. Only this time, he ate a larger amount of fava beans, which led to hemolysis and methemoglobinemia. Hence, while treating methemoglobinemia patients, one should be vigilant that a history of fava beans ingestion without any symptoms does not rule out G6PD deficiency. It depends upon the number of beans ingested over a certain period.^20^

## 5 CONCLUSION

Favism is a rare cause of the co‐occurrence of methemoglobinemia and hemolysis in G6PD deficient individuals. The severity of hemolysis in G6PD deficient individuals is dependent on the number of fava beans ingested. It is vital to identify the presence of G6PD deficiency in patients presenting with methemoglobinemia, as the initiation of methylene blue in such individuals can result in a cascade of oxidative hemolysis.

## CONFLICT OF INTEREST

All authors declare no potential conflicts of interest to disclose related to the publication of this case series.

## AUTHOR CONTRIBUTIONS

FA, BM: Conceptualized the data. BM: Consented the patient. FA, AB, SJ, ID, MM, MU, MY: Reviewed the literature. FA, BM, AB, SJ, ID: Wrote the manuscript. FA, AB: Collected the data. AB: Took part in writing and images radiologically. FA, MU, MY: Modified and critically reviewed the article. FA, AB, SJ, ID, MM, MU, MY: Reviewed finally and approved the article.

## CONSENT

Written informed consent was obtained from the patient for publication of this case report and accompanying images.
